# Somatic estrogen receptor α mutations that induce dimerization promote receptor activity and breast cancer proliferation

**DOI:** 10.1172/JCI163242

**Published:** 2024-01-02

**Authors:** Seema Irani, Wuwei Tan, Qing Li, Weiyi Toy, Catherine Jones, Mayur Gadiya, Antonio Marra, John A. Katzenellenbogen, Kathryn E. Carlson, Benita S. Katzenellenbogen, Mostafa Karimi, Ramya Segu Rajappachetty, Isabella S. Del Priore, Jorge S. Reis-Filho, Yang Shen, Sarat Chandarlapaty

**Affiliations:** 1 Human Oncology and Pathogenesis Program, Memorial Sloan Kettering Cancer Center, New York, New York, USA.; 2 Department of Electrical and Computer Engineering, Texas A&M University, College Station, Texas, USA.; 3 Department of Chemistry and Molecular and Integrative Physiology, and the Cancer Center, University of Illinois Urbana-Champaign, Urbana, Illinois, USA.; 4 Department of Pathology, Memorial Sloan Kettering Cancer Center, New York, New York, USA.; 5 Department of Computer Science and Engineering and; 6 Institute of Biosciences and Technology and Department of Translational Medical Sciences, College of Medicine, Texas A&M University, Houston, Texas, USA.; 7 Weill Cornell Medical College, New York, New York, USA.

**Keywords:** Endocrinology, Oncology, Breast cancer, Drug therapy, Sex hormones

## Abstract

Physiologic activation of estrogen receptor α (ERα) is mediated by estradiol (E2) binding in the ligand-binding pocket of the receptor, repositioning helix 12 (H12) to facilitate binding of coactivator proteins in the unoccupied coactivator binding groove. In breast cancer, activation of ERα is often observed through point mutations that lead to the same H12 repositioning in the absence of E2. Through expanded genetic sequencing of breast cancer patients, we identified a collection of mutations located far from H12 but nonetheless capable of promoting E2-independent transcription and breast cancer cell growth. Using machine learning and computational structure analyses, this set of mutants was inferred to act distinctly from the H12-repositioning mutants and instead was associated with conformational changes across the ERα dimer interface. Through both in vitro and in-cell assays of full-length ERα protein and isolated ligand-binding domain, we found that these mutants promoted ERα dimerization, stability, and nuclear localization. Point mutations that selectively disrupted dimerization abrogated E2-independent transcriptional activity of these dimer-promoting mutants. The results reveal a distinct mechanism for activation of ERα function through enforced receptor dimerization and suggest dimer disruption as a potential therapeutic strategy to treat ER-dependent cancers.

## Abstract 2

A set of naturally occurring mutations in the estrogen receptor promote ER dimerization and lead to constitutive activity and breast cancer cell growth.

## Introduction

Estrogen receptor α (ERα; *ESR1*), a member of the steroid hormone receptor family, is a hormone-activated transcription factor that is essential for both physiologic mammary gland homeostasis and pathologic breast tumor development (1, 2). Estrogen interacts with residues within the ligand-binding domain (LBD) of ERα to induce conformational changes that promote the binding of transcriptional coactivators, thereby activating its function (3–5). Point mutations in the LBD have been identified in over 35% of patients with anti-estrogen–resistant, metastatic breast cancer, with many of the mutations functioning to drive a nominally inactive apo-structure of ERα into one that mimics the estrogen-bound state (6–14). Approximately 20% of these clinically identified recurrent mutations, however, appear to have distinctive biologic activities and are located spatially far away from the other recurrent mutations that center on the loop between helix 11 (H11) and H12. Importantly, many of these mutations have been shown to drive hormone-independent transcription, suggesting they may be mimicking alternative modes of ERα activation (15).

In this report, we investigate the mechanisms by which mutations clustered around the ERα dimerization interface may promote conformational changes that enhance dimer stability and thereby support hormone-independent transcriptional functions. Drug inhibition studies and transcriptomic and other phenotypic assays demonstrate these mutants to be distinct from the previously characterized H12 mutants. Using a data-driven machine learning approach, we generated a mechanistic hypothesis about which conformational features differentiate these mutants from those previously described as regulating the position of H12. We establish that recurrent mutations in S463, V422, G442, F461, and L469 all localize near the dimer interface, promote dimer formation in vitro and in cells, and drive hormone-independent transcription and breast cancer growth in a manner that depends on dimerization. We further demonstrate that disruption of dimer formation suppresses the activity of these mutants. These data establish dimer augmentation as a biologically relevant mechanism of ERα activation and point to disruption of dimerization as a potential therapeutic strategy for cancers dependent on ERα function.

## Results

### A distinct class of activating ESR1 mutations.

**Figure 1:**
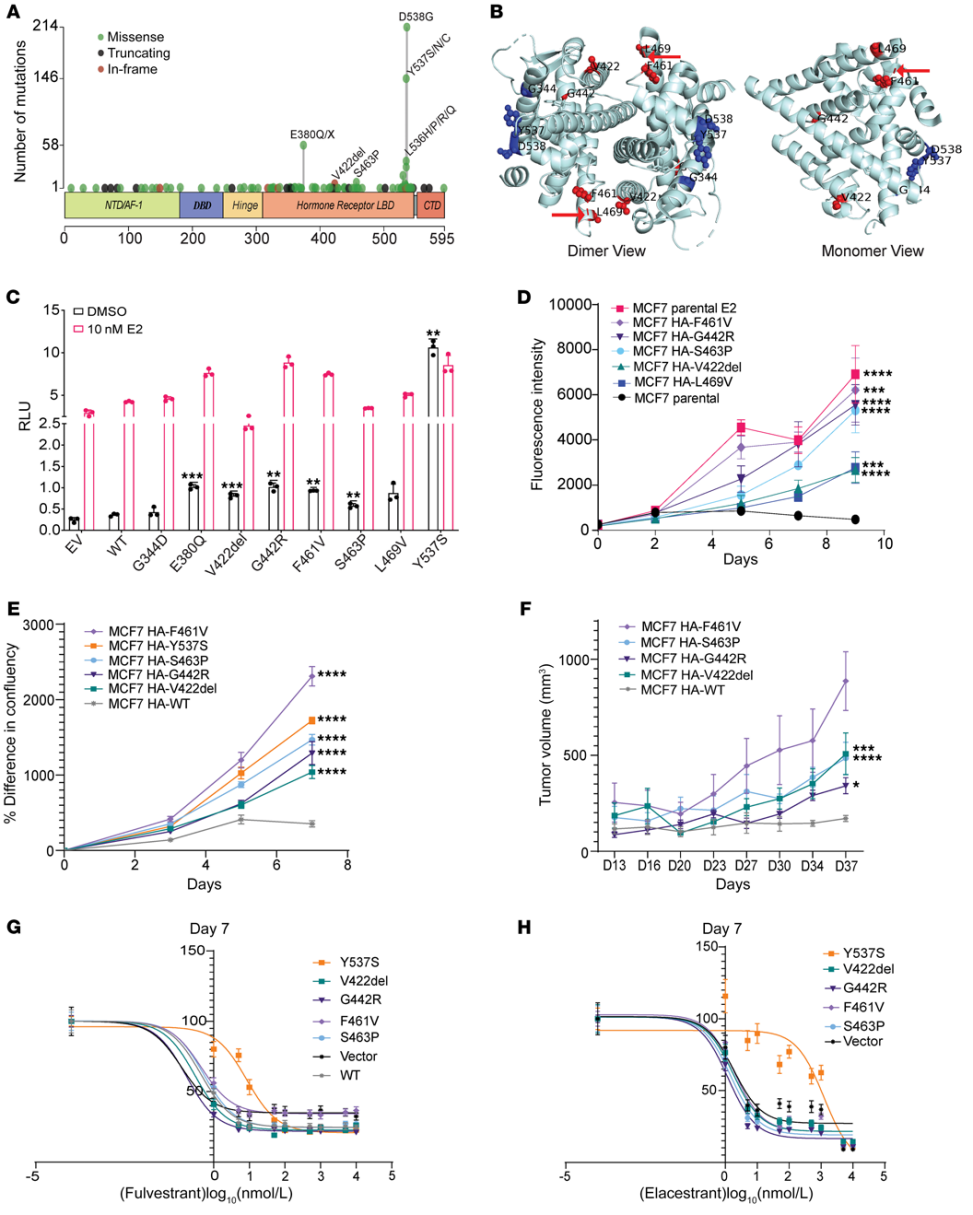
Activating *ESR1* mutations outside helix 12 of the LBD. (**A**) *ESR1* mutations (*n* = 649) in breast cancer samples from the MSK clinical sequencing cohort. NTD, N-terminal domain; DBD, DNA-binding domain; LBD, ligand-binding domain; CTD, C-terminal domain. (**B**) Mapping of a few of the *ESR1* mutations onto the structure of the ERα-LBD (Protein Data Bank ID 1GWR). Residues in the vicinity of the dimer interface are shown in red (a red arrow indicates the disordered region having residue S463), and those closer to the H11–H12 loop are shown in blue. (**C**) Luciferase reporter assay of MCF7 cells transfected with the HA-tagged *ESR1* mutants/WT or empty vector (EV), estrogen response element (ERE)–luciferase reporter, and *Renilla* luciferase reporter plasmids. The graph represents individual data points and mean ± SD (*n* = 3), with *P* values (Welch’s *t* test) for mutant versus WT indicated. (**D**) Cell viability of doxycycline-inducible (Dox-inducible) MCF7 cells and parental cells with or without 10 nM E2 growing in hormone-depleted medium supplemented with 0.5 μg/mL Dox; plotted as mean ± SD (*n* = 6), with *P* values calculated from Welch’s *t* test for mutant versus parental cells on the final day indicated. (**E**) Plot of the percentage increase in confluence from initial time point for the Dox-inducible MCF7 cells, growing in hormone-depleted medium with 0.5 μg/mL of Dox. Data are plotted as mean ± SEM (*n =* 6); statistical analysis was performed using 2-way ANOVA for mutant versus WT on the final day indicated. (**F**) MCF7 HA-ESR1 WT or mutant–expressing cell-derived xenograft tumor growth represented as mean ± SEM (*n =* 3–6 mice per group). The estrogen pellet was removed after the tumor volume reached 250 mm^3^, and mice were fed with Dox to induce HA-tagged ESR1 expression. Statistical analysis was performed using 2-way ANOVA on the final day indicated. F461V had 2 mice after day 16 and hence was not included in statistical analysis. (**G** and **H**) Growth inhibition of MCF7 cells expressing HA-ESR1 mutants or empty vector, as measured by cell viability assay, in the presence of fulvestrant (**G**) and elacestrant (**H**); EC_50_ values for sigmoidal fit are presented in Supplemental Tables 2 and 3. *P < 0.05, **P < 0.01, ***P < 0.001, ****P < 0.0001.

To assess for potential gain-of-function alterations affecting *ESR1*, we surveyed the Memorial Sloan Kettering (MSK) clinical sequencing breast cancer cohort, identifying 649 mutations in the LBD out of 8,302 samples analyzed (Figure 1A and Supplemental Table 1; supplemental material available online with this article; https://doi.org/10.1172/JCI163242DS1). Of these mutations, 471 (73%) correspond to well-established activating mutations in the loop between H11 and H12 (D538, Y537, L536), whereas 27% of mutations are observed outside of this region. While the mechanisms by which mutations in the loop between H11 and H12 function have been biochemically and structurally defined, the mechanisms of many of these other mutations have not. We mapped these other alterations onto the existing structures of 17β-estradiol (E2)-bound LBD and noted that the localization of several mutations fell within about 15 Å of the dimerization interface (Figure 1B). We selected a subset of these mutations (V422del, G442R, F461V, and S463P) along with L469V (reported previously, ref. 15) to investigate whether these might be mechanistically distinct from H12 mutants.

To determine the functional significance of these alterations and compare them with H11/H12 loop mutations, we transiently expressed them in hormone-dependent, ER^+^ breast cancer cells (MCF7 Tet-On) and evaluated their ability to stimulate transcription from an estrogen response element (ERE) reporter in the absence or presence of E2 (Figure 1C). In the absence of E2, we observed increases in E2-independent transcription from V422del (2.2-fold), G442R (2.8-fold), F461V (2.6-fold), S463P (1.6-fold), and L469V (2.4-fold) compared with wild type (WT). To evaluate the contribution of these mutants to breast cancer proliferation, HA-tagged versions of the mutants were conditionally expressed under a Tet promoter in 2 different ER^+^ breast cancer cell lines (MCF7 and T47D) (Supplemental Figure 1, A–C). Expression of the mutants facilitated estrogen-independent proliferation of the models to different levels when compared with parental cells or those transfected with an empty vector control, as observed from cell viability assays (Figure 1D and Supplemental Figure 1D). Cell confluence measurements over time also depicted an increase in proliferation for most mutants, a feature similar to that observed in the previously characterized H11/H12 loop mutation Y537S (Figure 1E). T47D cell line models also show reduced senescence (as observed from β-galactosidase staining) upon induction of mutant expression (Supplemental Figure 1E) under E2 depletion conditions, which aligns with these observations of increased cellular growth and expansion. To extend these findings in vivo, we prepared xenografts from the MCF7 cell lines expressing these mutants under a doxycycline-inducible promoter. Upon doxycycline treatment and removal of exogenous E2 supplementation, tumors expressing the V422del, G442R, F461V, or S463P mutant ER (Supplemental Figure 1F) showed significantly faster growth when compared with WT, further confirming their estrogen-independent activities (Figure 1F).

Previous work on the H12 mutant Y537S has demonstrated that cell lines expressing this mutant show partial resistance to selective estrogen receptor degraders (SERDs), such as fulvestrant, and selective estrogen receptor modulators (SERMs), such as tamoxifen (15–17). We compared the effect of such inhibitors on the proliferation driven by these recently characterized mutants using 3 different SERDs: fulvestrant (Figure 1G, Supplemental Table 2, and Supplemental Figure 1G), the recently approved first orally bioavailable SERD elacestrant (Figure 1H, Supplemental Table 3, and Supplemental Figure 1H) (18), and camizestrant (Supplemental Figure 1, I and J); and 2 SERMs: tamoxifen (Supplemental Figure 1K) and raloxifene (Supplemental Figure 1, L and M). Unlike with Y537S, cells with V422del, G442R, F461V, and S463P mutations retained comparable sensitivity to these antagonists compared with cells with WT ER.

Together, the data identify multiple, recurrent somatic mutations localized near the dimer interface of ERα that promote estrogen-independent ER-transcriptional activation and breast cancer growth.

### Machine learning models to elucidate mechanisms of ERα activation.

**Figure 2:**
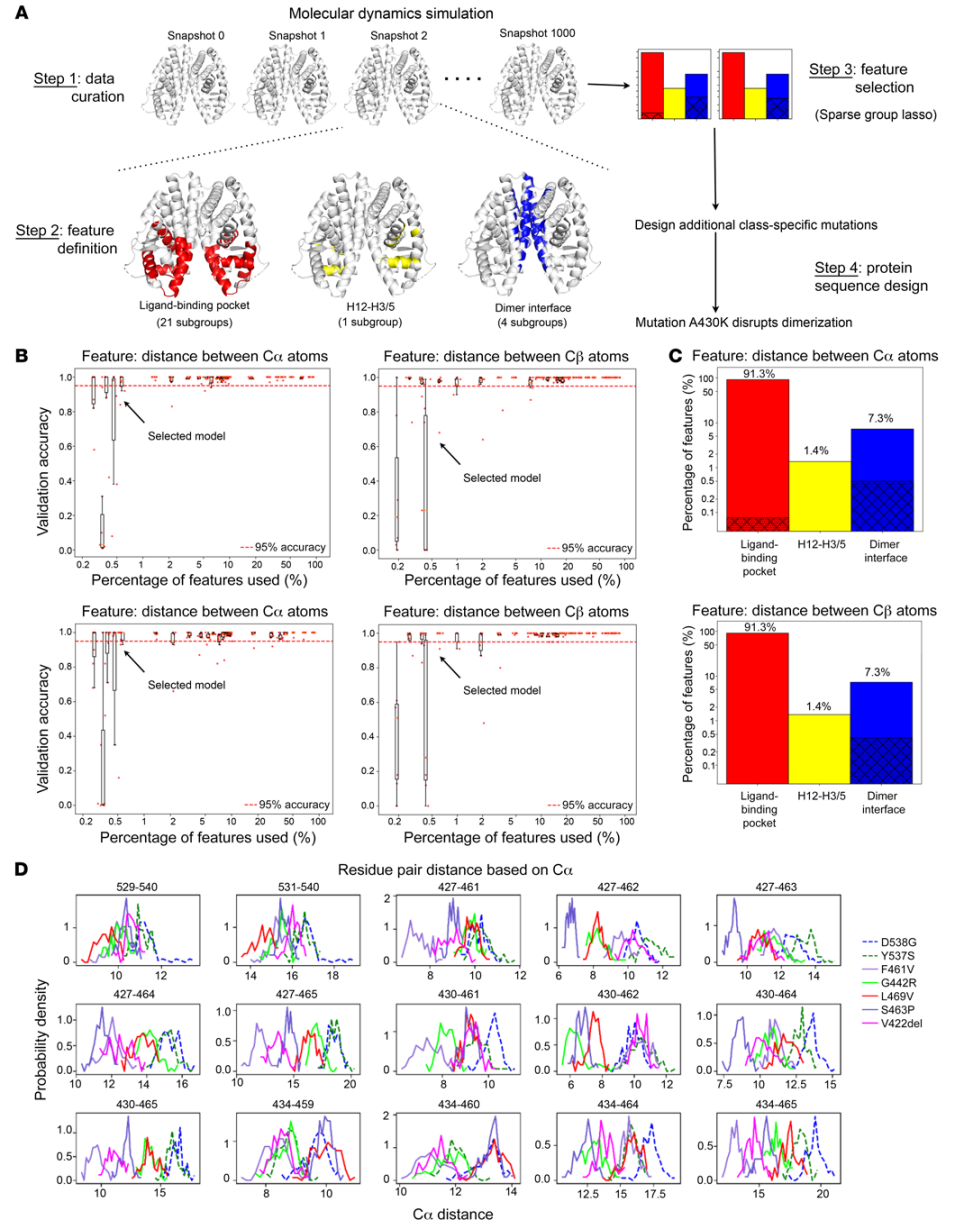
Developing machine learning models to simultaneously classify ERα-activating variants and select differential conformational features. (**A**) Flowchart representing the methodology adopted to study the differences between the 2 classes of *ESR1* mutations. Class I variants include Y537S and D538G, and class II variants include V422del, G442R, F461V, S463P, and L469V. (**B**) The accuracy versus percentage of features used, for the logistic regression models with sparse group lasso trained on 7 variants’ molecular dynamic trajectories (training set: 300 equi-spaced snapshots at 50–80 nanoseconds for each variant) to classify 7 activating variants into 2 classes, for the validation set (100 snapshots at 80–90 nanoseconds) (top) and the test set (100 snapshots at 90–100 nanoseconds) (bottom). On the left, features refer to the distances between the Cα atoms of respective residues; on the right, features refer to the distances between the Cβ atoms of residues. (**C**) Bar graphs showing the distribution of features across different regions of the ERα structure. In red are features that describe the geometry of the ligand-binding pocket (averaged over 2 monomers), in yellow are distances between H12 and surrounding H3/H5 (averaged over 2 monomers), and in blue are features that represent the monomer-monomer proximity across the dimer interface. The features selected by machine learning models (having over 95% median accuracy in classifying the variants) are shown as shadowed portions. On the top, features refer to the distances between the Cα atoms of respective residues; on the bottom, features refer to the distances between the Cβ atoms of residues. (**D**) Distributions of machine learning–selected pairwise Cα distances for 7 variants in class I (dashed line) and class II (solid line).

To gain insights into the variety of mutants localized to different regions of the LBD, we modeled their atom-level structures using molecular dynamics. Mutants were classified based on their location in the 3D structure — class I at/around H12: Y537S and D538G; and class II: V422del, G442R, F461V, S463P, and L469V, which, as described earlier, are close to the dimer interface. Specifically, we introduced each of these 7 activating variations separately to an x-ray crystal structure of E2-bound, WT ERα LBD and performed 100-nanosecond explicit-solvent molecular dynamics (MD) without E2, leading to snapshots 0–1,000 at an interval of 0.1 nanosecond for each variant. By splitting snapshots 501–1,000 into the training (501 to 800), validation (801 to 900), and test (901 to 1000) sets, we trained a logistic regression model with sparse group LASSO to classify the 2 types of MD snapshots while simultaneously choosing a small subset of features (Figure 2A). The original pool of features was 2,631 pairwise distances in 26 groups, between residues in 3 regions (Supplemental Figure 2A): 2,403 intrachain distances in 21 groups at the ligand-binding pocket (as in the agonist state), 36 intrachain distances in 1 group between H12 and H3/H5 (as seen in the antagonist state), and 192 interchain distances in 4 groups at the dimer interface.

The trained classifiers reached high accuracy by using a small number of conformational features that may mediate the basis for activation for the 2 classes of activating mutants. Whereas adding more features improved the classification accuracy, as few as 15 Cα-Cα distances (or 11 Cβ-Cβ ones) resulted in greater than 95% median classification accuracy for both the validation and the test sets over all variants (Figure 2B). This level of accuracy persisted when evaluated for individual variants’ validation and test sets as well (Supplemental Table 4). Even though 91.3% of the original 2,631 features belonged to the ligand-binding pocket and only 7.3% were at the dimer interface, 13 of the 15 pairwise distances between Cα atoms (or 11 of the 11 pairwise distances between Cβ atoms) selected by the machine learning models localized to the dimer interface (Figure 2C). Thus, the conformational features at the dimer interface are highly represented in the selected features, suggesting that the dimer interface is a major structural determinant differentiating the 2 types of activating variants.

The regression coefficients for these selected features were negative in most instances, suggesting that the corresponding cross-chain residue pairs moved closer together for the class II variants than for class I (Supplemental Figure 2B). A closer and more detailed comparison of the inter-residue distances of these selected features revealed that in the class II variants, the H8 residues at the dimer interface (427, 430, and 434) tended to be closer to the loop between H9 and H10 (462, 464, and 465) across the dimer interface (Figure 2D and Supplemental Figure 2C), potentially strengthening the interaction between these residues.

### Activating ESR1 mutations that promote receptor dimerization, dimer stability, and nuclear localization.

**Figure 3:**
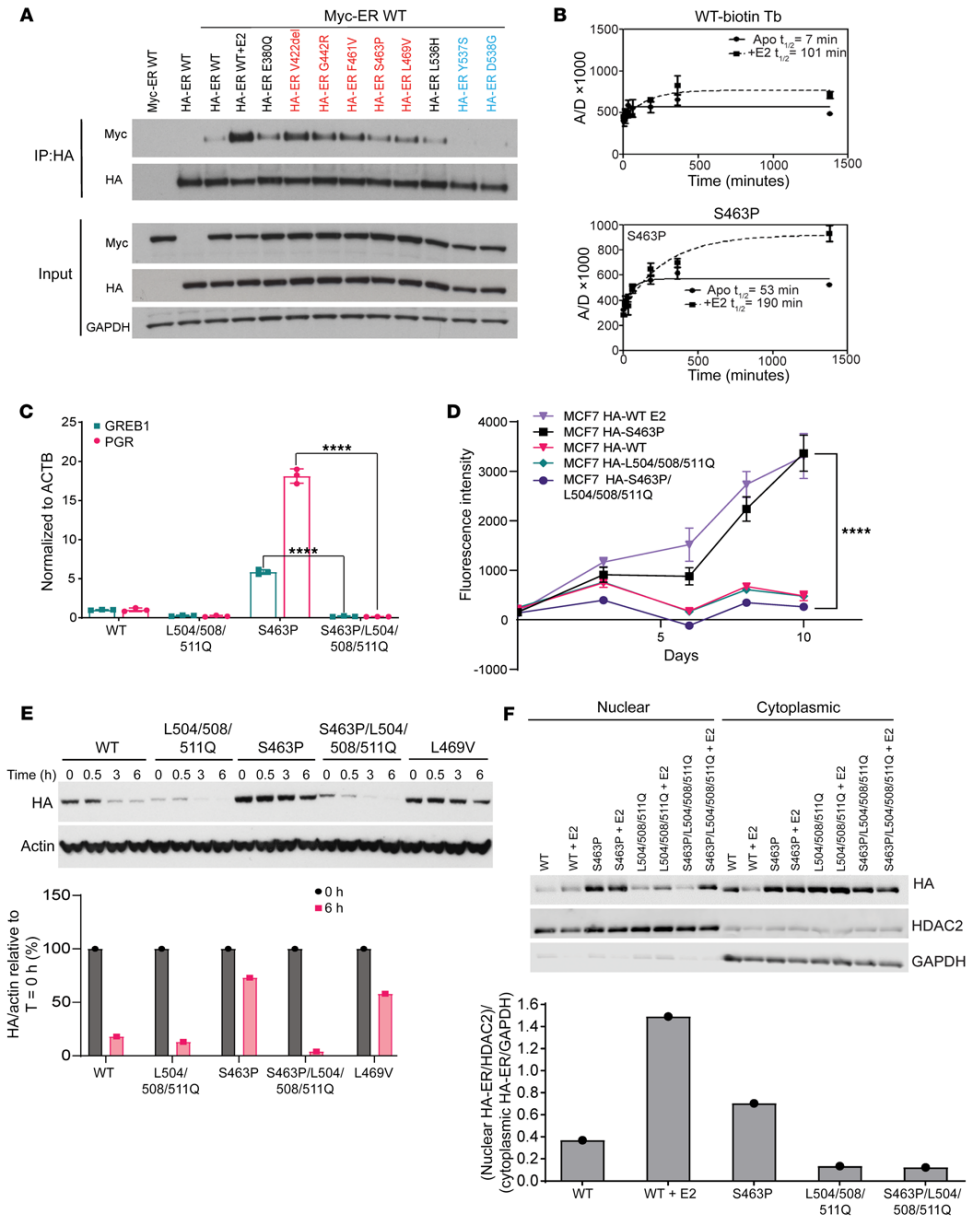
A distinct class of mutants that relies on dimerization for hormone-independent activity. (**A**) Immunoprecipitation of HA-ER WT/mutant from MCF7 cell lysate after cotransfection with plasmids containing MYC-ESR1 WT and HA-ESR1 WT/mutant. Class II mutants are shown in red, class I mutants in blue; 10 nM E2 was added wherever indicated. (**B**) Stability of WT ERα and S463P ERα LBD dimers evaluated by measurement of tr-FRET assay signal from dimer exchange of terbium-labeled ERα LBD at C417 and fluorescein-labeled ERα LBD at C530. The solid line indicates signal from the apo form of protein, and the dotted line indicates signal from protein exposed to 1 μM E2 for 30 minutes before dimer exchange. A/D, Acceptor emission/Donor emission. (**C**) Quantitative reverse transcription PCR (RT-qPCR) of GREB1 and PGR transcripts after growing of SKBR3 cells transiently transfected with HA-ESR1 WT or mutant plasmids in hormone-depleted medium. Bar graph/data points represent fold change relative to WT; error bars represent SD (*n =* 3 qPCR reactions); statistical analysis performed using 1-way ANOVA. (**D**) Cell viability of Dox-inducible HA-ER mutant/WT–expressing MCF7 cells growing in hormone-depleted medium with Dox; 10 nM E2 was added wherever indicated. Data are plotted as mean ± SD (*n =* 6); statistical analysis performed using 2-way ANOVA for the final day indicated. (**E**) Top: Immunoblotting of the HA-tagged ERα variants and actin levels from MCF7 cells growing in hormone-depleted medium, transiently transfected with the respective HA-ESR1 mutant plasmids, and exposed to HSP90 inhibitor SNX2112 at 500 nM concentration for indicated periods of time. Bottom: Signal quantification showing the ratio of HA to actin signal for *T =* 0 hours and *T =* 6 hours; signal ratio at *T =* 0 hours for each variant has been scaled to 100%. (**F**) Top: Immunoblotting of HA-tagged ERα variants in nuclear and cytoplasmic fractions of transiently transfected MCF7 cells that had been grown in hormone-depleted medium supplemented with 10 nM E2 whenever indicated. Bottom: Signal quantification showing relative enrichment of HA-ERα variants in the nucleus for samples in hormone-depleted medium. All densitometric analysis was performed on Image Studio Lite software. ****P < 0.0001.

Based on the location and predicted critical features of these activating class II mutants, we hypothesized that they promote the formation of dimers in the absence of E2. To first test for dimer stabilization, we assessed relative coimmunoprecipitation from MCF7 Tet-On cells transfected with HA-tagged WT or mutated ERα and Myc-tagged WT ERα growing in hormone-depleted medium (Figure 3A). We found that class II mutants, but not class I mutants, resulted in greater Myc pulldown of the coexpressed WT receptor (Figure 3A).

Two other mutations, L536H and E380Q, were also included in this assay based on their position in the ERα LBD structure (Supplemental Figure 3A). The L536H mutation, which can be thought of as an extension of class I mutants because of its close proximity to residues Y537 and D538, showed very weak dimerization, similar to that of WT ER without E2 (Figure 3A). We note that the third most common activating mutation in breast cancer is E380Q (Figure 1A); this mutation lies on H5 and is believed to favor the positioning of H12 by eliminating potential electrostatic repulsion (6). Furthermore, the E380 residue also lies within an approximately 15 Å radius from the dimer interface as the other class II mutation sites do. In these assays, it appeared to behave like members of the class II mutants, showing increased heterodimerization as compared with the class I mutants and WT (Figure 3A).

To further assess the enhancement in coimmunoprecipitation observed for class II mutants, we transfected MCF7 Tet-On cells with different ratios of HA-tagged ERα mutant and FLAG-tagged ERα WT. For the V422del mutation, we observed that expression of one-half of WT led to equivalent levels of coimmunoprecipitation, further supporting an enhanced dimerization potential for this mutant in the absence of E2 (Supplemental Figure 3B). We similarly observed enhanced (between 1.3- and 2-fold) dimerization from the L469V mutation (Supplemental Figure 3C).

To ascertain whether the cellular effects of mutant enhanced ER dimerization could be recapitulated in vitro, we assessed the stability of the S463P mutant using a previously described time-resolved Förster resonance energy transfer (tr-FRET) technique (19, 20). We observed that compared with WT ER, the S463P mutant in the absence of E2 showed substantially slower dissociation of ER dimers into monomers (7.6 times slower), indicating a much more stable dimer formation (Figure 3B).

This serves as strong evidence for the increased dimerization and stability of the class II mutants in the absence of E2, raising the question of whether these mutants depend on dimerization for their observed E2-independent activity. One way to test this is to disrupt dimerization and evaluate how it affects function. It has been shown previously that Leu504, Leu508, and Leu511 located on H11 are essential for receptor dimerization (21, 22). Thus, mutations in Leu504Gln/Leu508Gln/Leu511Gln (henceforth referred to as L504/508/511Q for brevity) were introduced into both the HA-ESR1 and HA-ESR1 S463P plasmids. Coimmunoprecipitation experiments demonstrated that the corresponding HA-ERα L504/508/511Q and HA-ERα S463P/L504/508/511Q manifested drastically reduced binding to FLAG-ERα WT even in the presence of E2 (Supplemental Figure 3D). This triple mutation of L504, L508, and L511 also showed no exchange signal in a tr-FRET experiment, in the absence or presence of E2, indicating no dimer formation (Supplemental Figure 3E). The lack of dimerization induced by this triple mutation blocked the estrogen-independent transcription driven by S463P (Figure 3C and Supplemental Figure 3F), and estrogen-independent breast cancer proliferation stimulated by S463P (Figure 3D and Supplemental Figure 3G).

ERα activation by ligand stimulation (23, 24) or class I mutation (16) promotes a receptor conformation that stabilizes the receptor and reduces its dependence on the HSP90 chaperone complex as a component of receptor activation. To ascertain whether class II mutants could similarly function to reduce HSP90 dependence, we evaluated the impact of HSP90 inhibition on mutant stability (Figure 3E). The level of HA-ERα fell below 20% for both the WT and the L504/508/511Q mutant as soon as 6 hours after HSP90 inhibitor treatment. By contrast, dimer-enhancing mutations S463P and L469V maintained expression levels well above 50% at these time points (Figure 3E). Moreover, addition of the L504/508/511Q mutation to S463P caused this slow degradation to become rapid, highlighting that the increased stability observed in these mutants was through the enhanced dimerization (Figure 3E and Supplemental Figure 3H).

Finally, in order for activated ERα to exert its key transcriptional functions, it must become localized to the nucleus, which occurs via nuclear localization sequences present in the hinge region between the DNA-binding domain (DBD) and the LBD (25). To assess the effect of dimer-promoting mutants upon the steady-state localization of ERα, we performed nuclear and cytoplasmic fractionation of MCF7 Tet-On cells transiently transfected with HA-tagged *ESR1* WT or mutant plasmids and probed for the levels of HA-tagged ERα present (Figure 3F). The ratio of the HA-tagged ERα in nuclear versus cytoplasmic fraction for the different mutants was plotted to gauge any relative differences in localization (Figure 3F). Consistent with its activating function, the S463P mutant resulted in an E2-independent increase in the nuclear/cytoplasmic ratio, which was eliminated when the L504/508/511Q mutation was additionally introduced (Figure 3F), correlating with the enhanced transcriptional activity for ER targets observed (Figure 3C).

Together, these data reveal that the activating class II mutants, being dimer inducing, depend on dimerization to overcome several of the barriers to ER activation, including HSP90 independence and nuclear localization.

### Selective disruption of enhanced dimerization impairs function.

**Figure 4:**
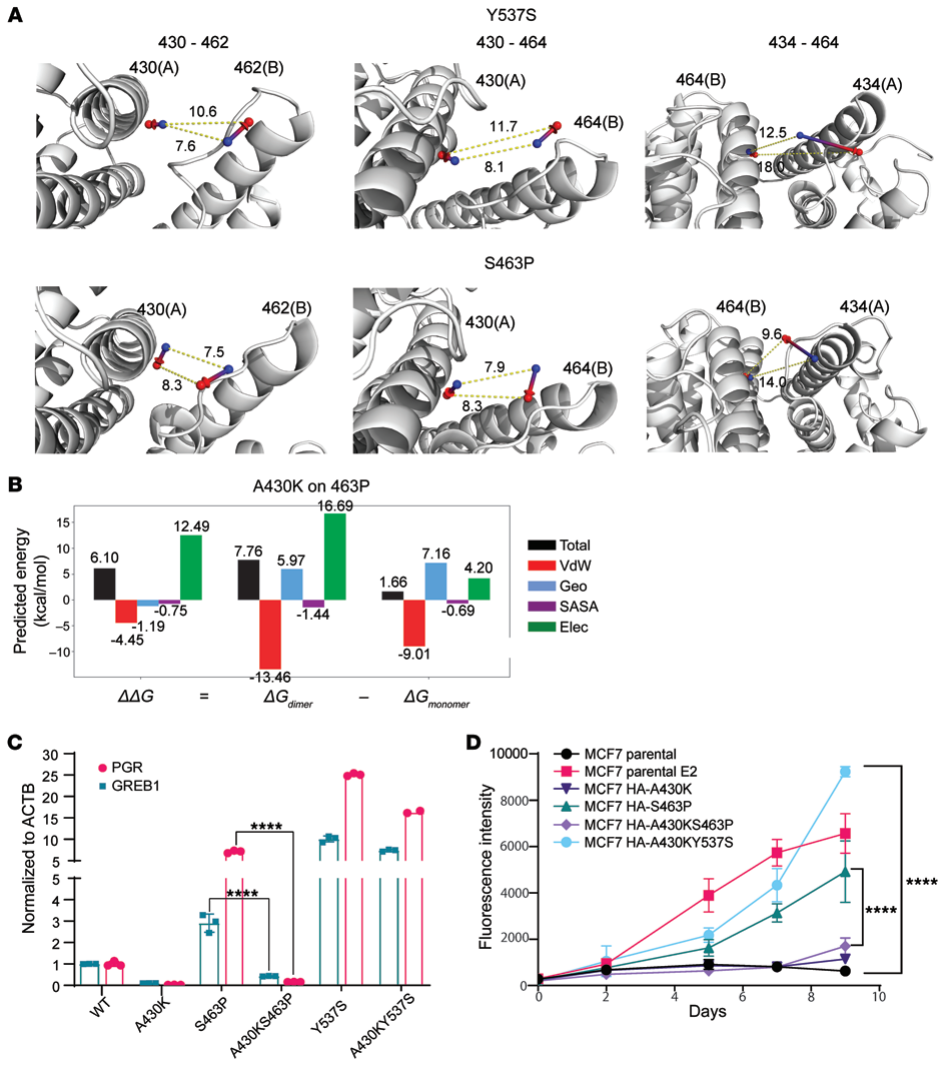
Selective disruption of enhanced dimerization impairs function. (**A**) Dynamics visualization of differential inter-residue Cβ distances selected by the activating-variant classifier (3 representatives: 430-462, 430-464, and 434-464). Arrow from blue to red spheres indicates the dislocation of corresponding Cβ atoms from 0 nanoseconds to 100 nanoseconds. (**B**) The secondary mutation of A430K to S463P was suggested by the multistate protein design method interconnected cost function networks (iCFN) to electrostatically weaken dimerization compared with S463P. The energy is decomposed into Van der Waals (VdW), geometric (Geo), solvent-accessible surface area (SASA), and electrostatics (Elec). (**C**) RT-qPCR demonstrating transcriptional activation of PGR and GREB1 genes in SKBR3 cells transfected with HA-ESR1 WT/mutant plasmids, grown in hormone-depleted medium for 48 hours. Bar graph represents fold change relative to WT, and error bars represent SD (*n =* 3 qPCR reactions); statistical analysis performed using 1-way ANOVA. (**D**) Cell viability assay of Dox-inducible MCF7 Tet-On stable cell lines expressing HA-tagged ER mutants, grown in hormone-depleted medium and supplemented with 0.5 μg/mL Dox. MCF7 Tet-On parental cell lines supplemented with 0.5 μg/mL Dox or 0.5 μg/mL Dox and 10 nM E2 are also included as controls. Data are plotted as mean ± SD (*n =* 6); statistical analysis performed using 2-way ANOVA for the final day indicated. ****P < 0.0001.

We next sought to selectively disrupt specific atomic interactions leading to enhanced dimerization of the class II mutants, in order to validate their contribution to the estrogen-independent activity observed. To identify residues specifically relevant to the enhanced dimerization, we compared the 15 machine learning–selected features across the last 10 nanoseconds of MD trajectories between Y537S, D538G, and S463P. Among these, the distinguishing and shorter Cα (backbone) distances across the dimer interface were found between residues 427/430 and 462 to 465 as well as between 434 and 464 (Figure 2D). Visualizing the distances between the Cβ atoms of 3 representative residue pairs, 430-462, 430-464, and 434-464, in the S463P and Y537S mutant structures observed in the MD simulations (Figure 4A) showed that, though the distance between the Cβ atoms at 0 nanoseconds was similar for both mutants, at 100 nanoseconds it was substantially different. The distance between Cβ atoms of residues 430 and 462 (or 464) was particularly distinct, being 6.4 Å (or 5.6 Å) shorter for the S463P mutant than for the Y537S mutant. Taken together, these results suggest that residues 430 and 434 and those on the loop between H9 and H10 are key contributors to the activating effects of S463P and candidate locations to disrupt such activating effects.

Interconnected cost function networks (iCFN) (26), a multistate protein design method, was used to design candidate mutations at each location for the variant S463P. Among all amino acid substitutions at residue 430, A430K, A430Y, and A430R were suggested by iCFN to disrupt dimerization for S463P the most while maintaining monomer folding, A430K being most effective of the three according to the predicted change in electrostatic binding energy (Figure 4B and Supplemental Figure 4A). We performed coimmunoprecipitation experiments using an A430K/S463P double mutation and confirmed that introduction of the A430K mutation disrupted dimerization, both in the absence and presence of E2 (Supplemental Figure 4B).

To assess the functional impact of the double mutant, we measured estrogen-independent expression of ER target genes induced by S463P (GREB1: 2.9-fold; PGR: 7.1-fold) compared with S463P/A430K (GREB1: 0.4-fold; PGR: 0.2-fold) and observed a complete block in the double mutant (Figure 4C). Moreover, expression of S463P in ER-dependent cells could support estrogen-independent growth, while the double mutant could not (Figure 4D and Supplemental Figure 4C). These results reveal that a mutation designed on the basis of a key differentiating feature of S463P can disrupt its ligand-independent activation. Furthermore, the effect of this mutation is specific to disrupting the activating effects of a class II mutant, since, when introduced together with a class I mutant (Y537S), it does not eliminate estrogen-independent transcription (Figure 4C), or proliferation (Figure 4D).

Taken together, the data reveal that selective disruption of dimerization can eliminate the ERα activation functions of class II mutants such as S463P, which validates the machine learning–predicted structural determinants by which S463P confers E2-independent activation.

## Discussion

Estrogen receptor activation is thought to be predominantly constrained by the activation energy required to induce a conformational change that promotes enhanced ERα nuclear localization and assembly of multiprotein complexes containing ERα that can promote transcription. The major stimulus that physiologically overcomes this activation energy is binding of the hormone estradiol. Several other forms of pathologic ERα activation have been observed, including somatic genetic mutations in the H11/H12 loop and posttranslational modifications such as phosphorylation (27–29). These alternative pathways to activation suggest additional restraints on ERα, some of which may ultimately be exploited through pharmacologic strategies that mimic or reinforce these restraints. We hypothesized that mining the full landscape of mutations in *ESR1* may lead us to such alternative pathways of activation.

In this study, we leveraged a cohort of more than 8,000 breast cancer patients whose cancers were subjected to multigene panel sequencing using the FDA-authorized Memorial Sloan Kettering Integrated Mutation Profiling of Actionable Cancer Targets (MSK-IMPACT) to identify the spectrum of *ESR1* mutations whose location and impact rendered them unlikely to simply mimic the immediate ligand-induced changes in the ligand-binding pocket. We identified several recurrent, somatic mutations in the region corresponding to the dimerization interface, all in patients with ER^+^ breast cancer and not observed in ER-negative cancers. To assess the mechanism by which these mutations function, we combined computational machine learning techniques with biochemical and cell biologic assays to establish that this set of mutations is distinct from the mutations in the H11/H12 loop and serves mainly to promote dimerization of the receptor. This enhanced dimerization leads to enhanced receptor stability, nuclear localization, transcriptional activation, and estrogen-independent breast cancer growth. The results point to another key constraint for ERα activation in its monomeric form and the therapeutic potential for disruption of ERα dimerization as an alternative or complementary strategy to antagonism of the ligand-binding pocket for the inhibition of ERα function. Regarding their sensitivity to ER antagonists, the mutants in this dimerizing class appear to be sensitive and do not seem to require substantially higher drug doses for complete inhibition as has been seen with the Y537S mutant. While some differences can be appreciated between different mutants or individual drugs (e.g., F461V and G442R for camizestrant), all dimer mutants are inhibited completely by fulvestrant across both MCF7 and T47D cell lines (Figure 1G and Supplemental Figure 1G), and no particular dimer mutant appears to be resistant to clinically relevant doses of the ER antagonists studied here.

Three key functional domains of ERα are the N-terminal domain (intrinsically disordered in conformation and regulating hormone-independent transcriptional activity), the DBD (which binds to estrogen-responsive DNA elements), and the LBD (containing the hormone-binding pocket, which interacts with the NR box motif of coregulators) (Figure 1A) (30). Residues of the LBD and DBD form the dimer interface of ERα, with H11, a portion of H8, and the loop between H9 and H10 of the LBD forming the majority of intermonomer contacts (3). The F-domain lying C-terminal to the LBD has been shown to reduce dimerization, and in vitro studies suggest a link between type of ligand (agonist, antagonist, and mixed agonist-antagonist) binding and dimer kinetic stability (20, 31). In this study we identify a set of mutations lying near or within the LBD dimeric interface that enhance dimerization of ERα in the absence of E2. The data supporting enhanced dimer stability include biochemical studies conducted in vitro and in cells. Three of these dimer-enhancing mutations lie along (F461V and S463P) or close to (L469V) the flexible loop formed between H9 and H10. We speculate that mutating S463 to proline may distort the backbone geometry and allow for more interactions with the opposite monomer, thereby stabilizing the LBD dimer.

As we observed that enhanced ERα dimerization was associated with ligand-independent ERα activity in breast cancer cells, we sought to establish its necessity using additional *cis*-acting mutations that could disrupt dimerization. First, using mutations that disrupt hydrophobic interactions along a large section of the dimer interface within H11, we established the general requirement for dimerization in receptor transcriptional function. Moreover, by computationally designing a mutation to specifically target residues that were predicted by machine learning to mediate class II mutant activation, we find that the enhanced dimer stability, which is a feature of these mutants, is indeed responsible for their ligand-independent activity. In both cases, it was possible to suppress ligand-independent dimer formation, which was associated with an abrogation of ERα activation for dimer-promoting mutants but not for H12 mutants. This abrogation of ERα activation includes blocking the core initial steps of ligand activation of the receptor, including its dissociation from HSP90 chaperone complexes and its shuttling from the cytoplasm to the nucleus (Figure 3, E and F). We speculate that dimerization may afford some of the features of stabilization brought about by HSP90 association, while potentially stabilizing the nuclear localization sequence in the hinge region to promote interactions with importins, thus contributing to the observed increase in nuclear enrichment.

The exact mechanism by which the more stable and nuclear-localized dimer mutants recruit transcriptional coactivators and activate transcription is unclear. It is tempting to suppose that the changes near the dimer interface can allosterically induce favorable positioning of H12, as hinted at by computational studies (32). Molecular dynamic simulations, albeit on monomers, have also predicted that the S463P mutation may increase the flexibility of H12 (33). In vitro tr-FRET experiments, however, have shown that in the absence of E2, the SRC3 nuclear receptor domain is unable to bind ER S463P LBD (15). While this would negate conventional recruitment of this coactivator protein, there are many ER-interacting proteins; and bringing two ER monomeric units together could possibly stimulate other types of interactions.

A recent paradigm posits that steroid hormone receptors form liquid-liquid phase-separated condensates at genomic loci. These are largely directed by weak multivalent interactions between intrinsically disordered regions (IDRs) found in the steroid hormone receptor N-terminal domains as well as in many recruited coactivators (34, 35). Indeed, it has recently been shown that E2 can stimulate ERα to enter into phase-separated assemblies with MED1 and components of the enhancer machinery at super-enhancers (6, 36–38). It is tempting to speculate that dimerization and subsequent increase in local concentration of IDRs allow these mutants to recruit coactivators despite the absence of E2.

Additional studies on the nuclear complexes and binding sites favored by distinct ER mutants will be of interest in establishing how these mutants may differentially become active. The findings overall suggest that highly distinct mechanisms of ER activation are being achieved by these distinct mutations, pointing to the potential for physiologic mimics of these distinct modes of ER activity that might be designed to block these distinct modes of activation. Perhaps certain biologic processes select for ER activation via the canonical ligand activation, leading to H12 movement, while other processes select for ER activation via dimerization (e.g., posttranslational modifications or protein-protein interactions). Further study of these distinct mechanisms should open insights into the protean processes under ER control within distinct cell types and developmental conditions.

## Methods

### Clinical samples from MSK-IMPACT cohort

We retrospectively surveyed all breast cancer specimens (*N* = 8,302) that underwent prospective genomic profiling using the FDA-authorized MSK-IMPACT multigene panel as previously described (39, 40) under Memorial Sloan Kettering Cancer Center (MSKCC) IRB protocol 12-245. After querying for cases carrying *ESR1* alterations, a total of 608 samples from 524 patients were identified. Pathology and clinical annotations were retrieved from medical charts. A list of all *ESR1* alterations included in this cohort is provided in Supplemental Table 5.

### Materials and reagents

17β-Estradiol (E2) and SNX2112 were obtained from Sigma-Aldrich and Pfizer, respectively. Primary antibodies used were from Cell Signaling Technology: ERα (D8H8) rabbit mAb (catalog 8644), HA tag (C29F4) rabbit mAb (catalog 3724), HA tag (6E2) mouse mAb (catalog 2367), Myc tag (71D10) rabbit mAb (catalog 2278), DYKDDDDK tag antibody (binds to the same epitope as Sigma-Aldrich’s Anti-FLAG M2 Antibody) (catalog 2368), β-actin (13E5) rabbit mAb (catalog 4970), and GAPDH (14C10) rabbit mAb (catalog 2118). Estrogen receptor α Monoclonal Antibody SP1 was obtained from Thermo Fisher Scientific (catalog MA5-14501). Secondary antibodies used for Western blot were anti-rabbit IgG (whole molecule)–peroxidase antibody produced in goat (Sigma-Aldrich, catalog A4914), anti-mouse IgG (GE Healthcare Bio-Sciences, catalog NXA931), and IRDye-labeled antibodies (LI-COR Biosciences, catalog 926-68071, catalog 926-32211, catalog 926-32210, catalog 926-68070). Anti-HA magnetic beads were obtained from Thermo Fisher Scientific (catalog 88837). All plasmids used were modifications of those described previously (16).

### Cloning and mutagenesis

The ESR1 gene was cloned into a pcDNA3.1(+) vector with an N-terminal 3XFLAG tag or 2XMyc tag using cloning procedures similar to those described previously (16). Various mutations were introduced in pcDNA-MYC-ERα, pcDNA-HA-ERα, and pSIN-TREtight-HA-ERα plasmids using the standard QuickChange method as described previously (15). The A430K mutation was introduced using PfuUltra II Hotstart PCR Master Mix (Agilent Technologies, catalog 600850) with primers designed to promote an exponential amplification (41).

### Cell culture

MCF7 Tet-On cells (also referred to as MCF7 cells in this article) were obtained from Clontech, T47D cells were obtained from American Type Culture Collection, and the SKBR3 and HEK293T cell lines were gifts from Neal Rosen (MSKCC, New York, New York, USA) and Ping Chi (MSKCC), respectively. These cell lines, all tested negative for mycoplasma, were cultured in DME/F12/NEAA (RPMI was used for T47D) with 10% FBS (Corning, reference 35-010-CV), 100 U/mL penicillin, 100 μg/mL streptomycin, and 2 mM l-glutamine in an incubator maintained at 37°C with 5% CO_2_. Short tandem repeat analysis was used to authenticate the MCF7 Tet-On and SKBR3 cell lines. T47D cell lines were transfected with a pInducer20 vector (pInducer20 was a gift from Stephen Elledge, Harvard Medical School, Boston, Massachusetts, USA, Addgene plasmid 44012) (42) and therefore contained rtTA to allow them to function as a Tet-On system.

Hormone-depleted medium was prepared by addition of 10% charcoal-stripped serum to phenol red–free DME/F12/NEAA (or RPMI for T47D) containing 100 U/mL penicillin, 100 μg/mL streptomycin, and 2 mM l-glutamine. Stable cell lines prepared from MCF7 Tet-On and T47D cells were maintained in medium prepared by use of Tet-free FBS (Takara Bio, catalog 631367) during regular passages.

### Transfections

All transfections were performed using the X-tremeGENE HP DNA transfection reagent (Roche, catalog 6366546001). Whenever cells grown in hormone-depleted medium were transfected, serum-free medium prepared using phenol red–free DME/F12/NEAA was used as a DNA diluent.

### Luciferase assay

Luciferase assays were performed as described previously (16); in brief, 0.2 × 10^6^ cells were seeded in each well of a 24-well plate. The next day, each of these wells was transfected with 0.125 μg of pcDNA-HA-ERα (WT or mutant), 0.315 μg of 3x-ERE-TATA-luciferase reporter, and 0.06 μg of pRL-TK *Renilla* luciferase plasmids. Approximately 24 hours later, E2 was added at a concentration of 10 nM for MCF7 Tet-On cells. After 24 hours of E2 stimulation, trays were taken out of the incubator, and the supernatant medium was discarded before each well was washed once with PBS. Then, reagents and standard protocols recommended by the Dual Luciferase Reporter Assay kit (Promega, catalog E1980) were used to measure the luminescent signal from the bioluminescent reactions catalyzed by the firefly and *Renilla* luciferases. All measurements were made on a Veritas Microplate Luminometer (Promega); RLU was calculated as the ratio of the firefly to the *Renilla* luciferase activity.

### Stable cell line generation

All stable cell lines were generated by retroviral infection, similar to the protocols described previously (16). Around 3 × 10^6^ HEK293T cells were seeded in 6 cm dishes with 5 mL of regular complete medium. A day after seeding, cells were transfected with 2.25 μg of PCL-AMPHO, 0.5 μg of pCMV VSVG, and 2.25 μg of pSIN-TREtight-HA-ERα plasmids in WT form or with the respective mutation introduced. Four to six hours later, the medium was replaced with regular complete medium prepared with Tet-free FBS for virus production. Forty-eight hours later, the medium from the cells was carefully removed and centrifuged, then passed through a 0.45 μm filter, and Polybrene (Santa Cruz Biotechnology, catalog sc-134220) was added to a final concentration of 8 μg/mL. MCF7 or T47D Tet-On cells seeded in T25 flasks at 1 million cells the previous day were infected by replacing of the medium with the preparation described above and incubating for about 28 hours. The cells were then transferred to T75 flasks and allowed to grow for 60–72 hours, before a selection in 500 μg/mL of Hygromycin B Gold (InvivoGen, catalog ANTHG5) was carried out for 2 weeks.

Unless stated otherwise, the MCF7 and T47D cells prepared as described above were exposed to 0.5 μg/mL and 1 μg/mL doxycycline, respectively, to induce expression of HA-tagged *ESR1* mutants and WT.

### Cell viability assays

Cells (1,500 per well) were seeded in 96-well plates in hormone-depleted medium in 6 replicates per sample. A final concentration of 0.5 μg/mL or 1 μg/mL of doxycycline was added to induce expression of HA-ERα WT or mutants under the Tet promoter. When E2 was used to stimulate cell proliferation, a final concentration of 10 nM E2 was used. Cells were grown in a humidified incubator maintained at 37°C with 5% CO_2_. Readings were taken at respective time points by addition of 25 μL of resazurin (R&D Systems, catalog AR002) to the 200 μL of contents present in each well and incubation at 37°C for 4 hours, followed by measurement of the fluorescence emission at 590 nm (excitation wavelength 560 nm) on a microplate reader (SpectraMax M5, Molecular Devices). Wells containing equivalent volume of medium (no cells) and resazurin were used as the plate blank. Raw values of fluorescence after correction with the plate blank values were plotted on GraphPad Prism.

### Cell confluence

MCF7 cells expressing either HA-ERα WT or mutants were plated in 96-well plates (Corning 3610) at 1,500 cells per well in regular medium. The next day the medium was replaced with hormone-deficient medium with 0.5 μg/mL final concentration of doxycycline to induce expression. Ten nanomolar E2 was added to the cells expressing HA-ERα WT. Confluence was analyzed on days 0, 3, 5, and 7 using IncuCyte Adherent cell-by-cell live cell imaging software. Cell confluence values over the various time points were normalized to day 0, and the percentage difference in confluence with time was plotted for the mutants and WT.

### Drug inhibition assays

Cells (1,500 per well) were seeded in 96-well plates in regular medium in the presence or absence of doxycycline. Cells were treated with different inhibitor concentrations the next day, and measurements of cell viability were made on the days mentioned using the methodology described earlier. Data points were plotted using GraphPad Prism and fit to a sigmoidal regression model; EC_50_ values were reported with the fitted graphs.

### Animal studies

All studies were performed at the Memorial Sloan Kettering Cancer Center in compliance with institutional guidelines under an Institutional Animal Care and Use Committee–approved protocol (MSKCC 12-10-016). Six- to eight-week-old NSG female mice procured from Harlan Laboratories were maintained in pressurized ventilated caging. Xenograft tumors were established by subcutaneous implantation of 0.18-mg sustained-release 17β-estradiol pellets with a 10 g trocar into one flank followed by injection of 10 million MCF7 HA-ER WT or mutant cells suspended 1:1 (volume) with reconstituted basement membrane (Matrigel, Collaborative Research) on the opposite side after at least 3 days. Three to six mice were in each group. When tumors reached a size of 250 mm^3^, the pellet was removed, and mice were fed with food containing doxycycline (Envigo, TD.01306). Tumor volumes and weights were measured twice every week, with the dimensions measured with Vernier calipers and tumor volumes calculated [π/6 × larger diameter × (smaller diameter)^2^]. Graphs were plotted in Prism with *P* values calculated from a 2-way ANOVA, controlling for multiple comparisons using FDR (2-stage step-up method of Benjamini, Krieger, and Yekutieli).

### β-Galactosidase staining

Cells (0.3 million) were seeded in 6-well plates, and 1 μg/mL doxycycline was added. Cells were fixed at day 5, and β-galactosidase staining was performed based on the manufacturer’s instruction (Cell Signaling Technology 9860). After staining, images were taken under a Nikon Eclipse Ti2 microscope using a DS-Fi3 camera.

### Coimmunoprecipitation

Coimmunoprecipitation experiments were performed by seeding of 10 cm dishes with 2–3 million cells. After a day of growth in hormone-depleted medium, cells were transfected with pcDNA HA-ESR1 plasmids (carrying respective mutations) using the X-tremeGENE HP DNA transfection reagent. Transfections were performed using standard manufacturer-recommended protocols, with serum-free medium prepared using phenol red–free DME/F12/NEAA as a DNA diluent. Approximately 24 hours after transfection, 17β-estradiol (E2) or vehicle (DMSO) was added as indicated. Forty-eight hours after transfection, the cells were washed twice with ice-cold PBS and harvested. The resulting cell pellet was then resuspended in lysis buffer (1% NP-40, 50 mM Tris-Cl [pH 7.5], 150 mM NaCl, 5 mM EDTA, supplemented with Halt protease and phosphatase inhibitor cocktail [Thermo Fisher Scientific] right before use) approximately 3–4 times its volume, before being placed on a rotator at 4°C for 30 minutes. The lysed suspension was then spun down at 15,000*g* for 15 minutes at 4°C to remove cell debris, and total protein concentration in the supernatant was measured using the Pierce BCA Protein Assay kit (Thermo Fisher Scientific, catalog 23225). Thirty to fifty micrograms of this lysate was run on a gel as total protein input. Five hundred micrograms of protein lysate diluted to a final concentration of 1 μg/μL in lysis buffer was incubated with 10 mg of anti-HA magnetic beads overnight at 4°C on a rotator, after which the beads were washed 4 times with 800 μL to 1 mL lysis buffer before elution in 1× LDS buffer (containing 10 mM DTT) at 70°C for 10 minutes. Samples of input and elution were then probed using standard immunoblotting techniques. To detect the levels of bait protein in the elution sample, the membrane was stripped using either Thermo Fisher Scientific Restore Plus Western Blot Stripping Buffer (catalog PI46430) or NewBlot Nitro Stripping Buffer (LI-COR Biosciences, catalog 928-40030) according to procedures recommended by the manufacturer.

### Immunoblotting

Cells were seeded in hormone-depleted medium. For expression tests, 0.5 μg/mL of doxycycline was added a day after seeding. For SNX2112 treatment, transient transfections were performed the day after seeding; around 24 hours later, cells were divided into several smaller dishes (to allow for different time periods of exposure to drug), and the indicated amount of drug was introduced after another 24 hours. At the time points specified, cells were harvested by decanting of the medium, washing twice with ice-cold PBS, and then scraping and collecting of microcentrifuge tubes. The cell pellet formed after centrifuging at 4,000*g* for 2 minutes at 4°C was lysed in RIPA lysis buffer supplemented with protease and phosphatase inhibitors using standard manufacturer-recommended protocols. The supernatant was separated from cell debris by centrifuging at 15,000*g* for 15 minutes at 4°C. A BCA assay was used to measure the total protein concentration, and an equal amount of protein was loaded in NuPAGE Bis-Tris gel (Thermo Fisher Scientific). After transfer and blocking, the membranes were exposed first to primary antibodies overnight at 4°C, and then to secondary antibodies labeled with either HRP or IRdye at room temperature. Imaging was performed either using chemiluminescent HRP substrate or on an Odyssey Imaging System (LI-COR Biosciences). All quantifications were performed using Image Studio Lite 5.2 (LI-COR Biosciences). All Western blots in the article represent 1 of a minimum of 2–3 independent repeated experiments.

Quantitative reverse transcription PCR Forty-eight hours after transient transfection of cells grown in hormone-depleted medium, RNA extraction was performed using the RNeasy Mini Kit (Qiagen, catalog 74106). Reverse transcription was performed on 500 μg of extracted RNA using the qScript cDNA SuperMix (VWR, catalog 101414-106). Quantitative PCR (qPCR) reactions were set up in triplicates with an amount of cDNA corresponding to 25 ng of the original RNA using the TaqMan Universal PCR Mastermix (Thermo Fisher Scientific, catalog 4364340) and TaqMan probes for GREB1 (Thermo Fisher Scientific, catalog Hs00536409_m1), PGR (Thermo Fisher Scientific, catalog Hs01556707_m1), and ACTB (Applied Biosystems, catalog 4352935E). Data were normalized to ACTB levels, and averages and standard deviations were calculated from triplicate qPCR reactions.

### Time-resolved Förster resonance energy transfer assay

To measure dimer exchange by time-resolved Förster resonance energy transfer (tr-FRET), 2 aliquots of ER-LBD were site-specifically labeled (20, 43), one at C417 with 30 equivalents of a thiol-reactive biotin derivative (Biotin-dPEG3-MAL, Quanta Biodesign), to which was added the donor fluorophore, a streptavidin-terbium chelate (SaTb, Invitrogen), and the other labeled at C530 with the acceptor fluorophore fluorescein (iodoacetamidofluorescein, Invitrogen). They were separately incubated with 1 × 10^–6^ M of ligand for 0.5 hours. The two ER-ligand complexes were mixed together at concentrations of 2 nM ER-SaTb and 10 nM ER-fluorescein, and aliquots were taken with time and measured for the development of the FRET signal. Individual aliquots were measured at each time point to avoid bleaching artifacts. As the dimers exchange, there is a development of the FRET signal only when dimers have both donor (terbium complex) and acceptor (fluorescein) fluorophores (20, 43). Measurements were performed, in 96-well black plates, with a PerkinElmer Victor X5 plate reader using an excitation filter at 340/10 nm and emission filters for terbium and fluorescein at 495/20 and 520/25 nm, respectively, with a 100-microsecond delay. Diffusion-enhanced FRET was determined by a parallel incubation without biotinylated ER-LBD and subtracted as a background signal.

### Nuclear and cytoplasmic extraction

MCF7 Tet-On cells grown in hormone-depleted medium for a day were transiently transfected with pcDNA HA-ERα WT or mutant plasmids. A day after transfection, cells were exposed to either 10 nM E2 or vehicle (DMSO) control for 24 hours and harvested. Nuclear and cytoplasmic components were isolated using the NE-PER Nuclear and Cytoplasmic Extraction Reagents kit (Thermo Fisher Scientific, catalog 78833) following the manufacturer-recommended protocols. Ten to fifteen percent (vol/vol) of the cytoplasmic and nuclear fractions were loaded onto a NuPAGE 4%–12% Bis-Tris gel, and standard immunoblotting techniques were used to determine the levels of HA-tagged ERα, HDAC2, and GAPDH. HDAC2 was used as a loading control for the nuclear fraction (44) and GAPDH for the cytoplasmic fraction (45). Image Studio Lite 5.2 was used to quantify the intensity of the bands seen, and the relative enrichment in the nucleus as compared with the cytoplasm was calculated as (Nuclear HA–ERα/nuclear HDAC2)/(Cytoplasmic HA–ERα/cytoplasmic GAPDH) and plotted in Microsoft Excel.

### Structural preparation and molecular dynamics

3D atomic structures of each ERα LBD variant were built by modification of an E2-bound WT dimer structure (Protein Data Bank ID 1GWR) using the molecular modeling program CHARMM (https://charmm.chemistry.harvard.edu). Each variant structure without E2 was subject to explicit-solvent molecular dynamics (MD) for 100 nanoseconds using the computer program NAMD (https://www.ks.uiuc.edu/Research/namd). More details about structural preparation under CHARMM and molecular dynamics under NAMD can be found in ref. 14.

### Machine learning

#### Data processing.

The 100-nanosecond MD trajectory of each variant was sampled every 0.1 nanoseconds into 1,000 snapshots. The last 500 snapshots (501 to 1,000) were regarded as the stage and split into training (501 to 800), validation (801 to 900), and test sets (901 to 1,000). The samples (snapshots) were regarded as effectively independent because consecutive snapshots were 0.1 nanoseconds apart, i.e., 50,000 steps apart in MD simulations with a time step of 2 femtoseconds.

#### Feature calculation.

For each conformational sample, we calculated a set of features that are defined as pairwise distances between residues (using coordinates of Cα or Cβ atoms) and are invariant to rotations or translations of the sampled structures. We focus on such features at 3 sites: (a) Within-chain agonist-state ligand-binding pocket. We selected 7 subsets of the residues corresponding to various patches of the pocket and calculated pairwise distances across the patches. Specifically, the 7 patches include part of H3 (residues 342–354), H6 (resi. 383–394), S1/S2 hairpin (resi. 402–410), H8 and the preceding loop (resi. 418–428 except 422 that was deleted in V422del), H11 (resi. 517–528), loop 11–12 (resi. 529–538), and H12 (resi. 539–547). There were 2,403 pairwise distances, in 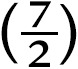 = 21 groups, defining the geometry of the agonist-state ligand-binding pocket. (b) Within-chain antagonist-state geometry involving pairwise distances between H12 (resi. 539–547) and its binding partners on H3/H5 (resi. 358, 372, 376, 380). There were 36 pairwise distances in 1 group defining the agonist-state H12 interactions. (3) Across-chain dimer interfaces including 4 groups: those between two H11 (resi. 497, 504, 505, 508, 509, 511, 512, 513, 515, 516, 519, 520, 523), between H9 (resi. 455, 456, 458, 459) and H11 (resi. 498, 501, 502, 505, 506, 509, 510, 513), between H10 (resi. 479, 480, 483, 484, 487) and H11 (resi. 498, 501, 502, 505, 506, 509, 510, 513), and between H8 (resi. 427, 430, 434) and the loop next to H9 (resi. 459, 460, 461, 462, 464, 465). There were 192 pairwise distances in 4 groups at the dimer interface. In total, we had 2,631 conformational features belonging to 26 groups of pairwise distances at 3 sites. Each distance was calculated twice due to the symmetry in the dimer and averaged. It was then standardized by subtracting its value in the initial snapshot of MD simulations and dividing its standard deviation across training snapshots of all 7 variants. As all variants shared very similar initial structures in MD simulations, a negative- or positive-valued feature indicates a closer or farther distance than that in the agonist-state WT.

#### Classification labels.

Each snapshot is classified according to the corresponding variant: class I for Y537S and D538G and class II for V422del, G442R, F461V, S463P, and L469V.

#### Model training.

For each sample *i*, given its 2,632-dimensional features *x_i_* (aforementioned 2,631 distances and a constant 1) and a binary label *y_i_* (0 for class I and 1 for class II), a logistic regression model assumes that the logit of the probability is linear in the features: logit(*P*(*y_i_* = 1 | *x_i_,β*)) = *β^T^x_i_*; in other words, *P*(*y_i_* = 1 | *x_i_,β*) is a logistic function *σ*(*β^T^x_i_*). To find the model parameters (feature coefficients *β*), the loss function for training the logistic model includes the weighted binary cross entropy as well as sparse group lasso. Specifically, the loss to minimize includes (a) the negative cross entropy
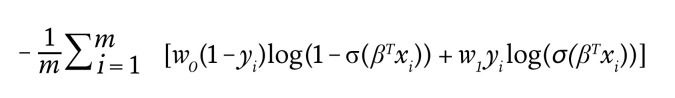


 Equation 1

where *w*_0_ and *w*_1_ are class weights; and (b) sparse group lasso regularization terms
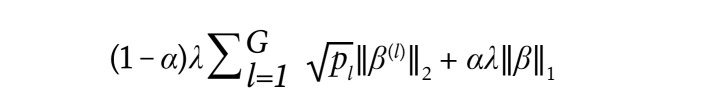


 Equation 2

where *l* is the feature group index, *G* is the total number of groups, *β*^(*l*)^ is the subvector of *β* corresponding to features in group *l*, *λ* controls the overall regularization strength, and *α* balances group sparsity and feature sparsity. Hyperparameters *α* and *λ* were searched over uniform grids {0, 0.1, 0.2, … , 1.0} and log-uniform grids {1 × 10^–4^, 1 × 10^–3^, 1 × 10^–2^, … , 1 × 10^3^}, respectively. For each hyperparameter combination, parameters *β* were trained over the training set using the Python Keras library. The classification accuracy over the validation set (across all 7 variants or for each individual variant) was used to tune the hyperparameters as well as the threshold for *β* to retain important features. Specifically, the resulting optimal hyperparameters *λ* and *α*, for the overall sparsity-regularization strength and balancing of inter- and intragroup sparsity, respectively, were set at 1 × 10^–4^ and 0.5, respectively (Supplemental Figure 5); and the optimal threshold for *β* was chosen to be 1 × 10^–1.6^ to balance the need to reduce features and the need to maintain model accuracy. Once features were selected, machine learning models were retrained without sparsity regularization, using the selected features alone, to assess their accuracy.

### Computational protein design

Our computational protein design approach designs experiments that can directly test the mechanistic hypotheses proposed by machine learning. Specifically, based on the machine learning–selected snapshots and a few important features, we select corresponding positions as candidates and design a secondary mutation to perturb the conformational features and ultimately to abolish the activating functionality of S463P.

We used interconnected cost function networks (iCFN) (26), a multistate protein design method. The positive state here is the dimer, and the negative state is the monomer. Both states were represented by an ensemble of 10 conformational structures (substates), namely, the top 10 test snapshots for S463P that were classified class II with the highest probabilities by the machine learning model. iCFN efficiently searches the sequence space to minimize the difference of the folding energy between the positive and the negative states, that is, the dimer binding energy while simultaneously minimizing the structures in both states and restricting the negative-state folding stability to be at most 5 kcal/mol worse than that of the WT. A low-resolution energy model used in this stage is the sum of 4 terms: internal (geometric) energy (Geo), Van der Waals (VdW), electrostatics (Elec) terms, and nonpolar contribution to solvation that is dependent on solvent-accessible surface area (SASA). The top structures of each designed top sequence variant are reevaluated and re-ranked in a high-resolution energy model where continuum electrostatics replaces Coulombic electrostatics in the low-resolution energy model. The energy difference between the dimer and the monomer state is termed the binding energy. The binding energy difference between a double-mutation variant and its “wild type” (S463P here) is decomposed into the 4 energy terms, where continuum electrostatics was focused on because of its relative insensitivity to small errors in structural modeling. More details can be found in ref. 26 and ref. 46.

### Statistics

All statistical tests used are identified in figure legends or Methods. Either 2-tailed Welch’s *t* test (unequal variances *t* test) or 1-way or 2-way ANOVA was used for statistical analysis. When 2-way ANOVA was used, Tukey’s or Dunnett’s multiple-comparison test was used, or multiple comparisons were corrected for by controlling of the FDR using the 2-stage step-up method of Benjamini, Krieger, and Yekutieli. Description of error bars is provided in the respective figure legends. Unless specified otherwise, all graphs were plotted on GraphPad Prism 9.3.1. *P* < 0.05 was considered significant.

### Study approval

Mouse studies for Figure 1F and Supplemental Figure 1F were performed at the Memorial Sloan Kettering Cancer Center in compliance with institutional guidelines under an Institutional Animal Care and Use Committee–approved protocol (MSKCC 12-10-016). Figure 1A and Supplemental Tables 1 and 5 were prepared following a retrospective analysis of clinical samples from the MSK-IMPACT cohort.

### Data availability

Data sets used to prepare the plots in this article can be found in the Supporting Data Values spreadsheet.

## Author contributions

SI, W Toy, QL, YS, and SC designed the research studies. AM and JSRF performed analysis of clinical samples. W Tan, MK, and YS performed the machine learning and computational studies. JAK, KEC, and BSK performed the tr-FRET experiments. SI, QL, W Toy, CJ, MG, RSR, and ISDP performed all the rest of the experimentation and data analysis. SI, QL, W Tan, YS, and SC wrote the manuscript with input from all authors.

## Supplementary Material

Supplemental data

Supplemental table 5

Supporting data values

## Version 1 10/26/2023

In-Press Preview

## Version 2 01/02/2024

Electronic publication
