# Supplementary material for: Somatic estrogen receptor α mutations that induce dimerization promote receptor activity and breast cancer proliferation
Source: J Clin Invest. 2024 Jan 2;134(1):e163242. doi: 10.1172/JCI163242 (PMC10760953; doi:10.1172/JCI163242)
Supplement: Supplemental data [file jci-134-163242-s230.pdf]

Supplementary Figures

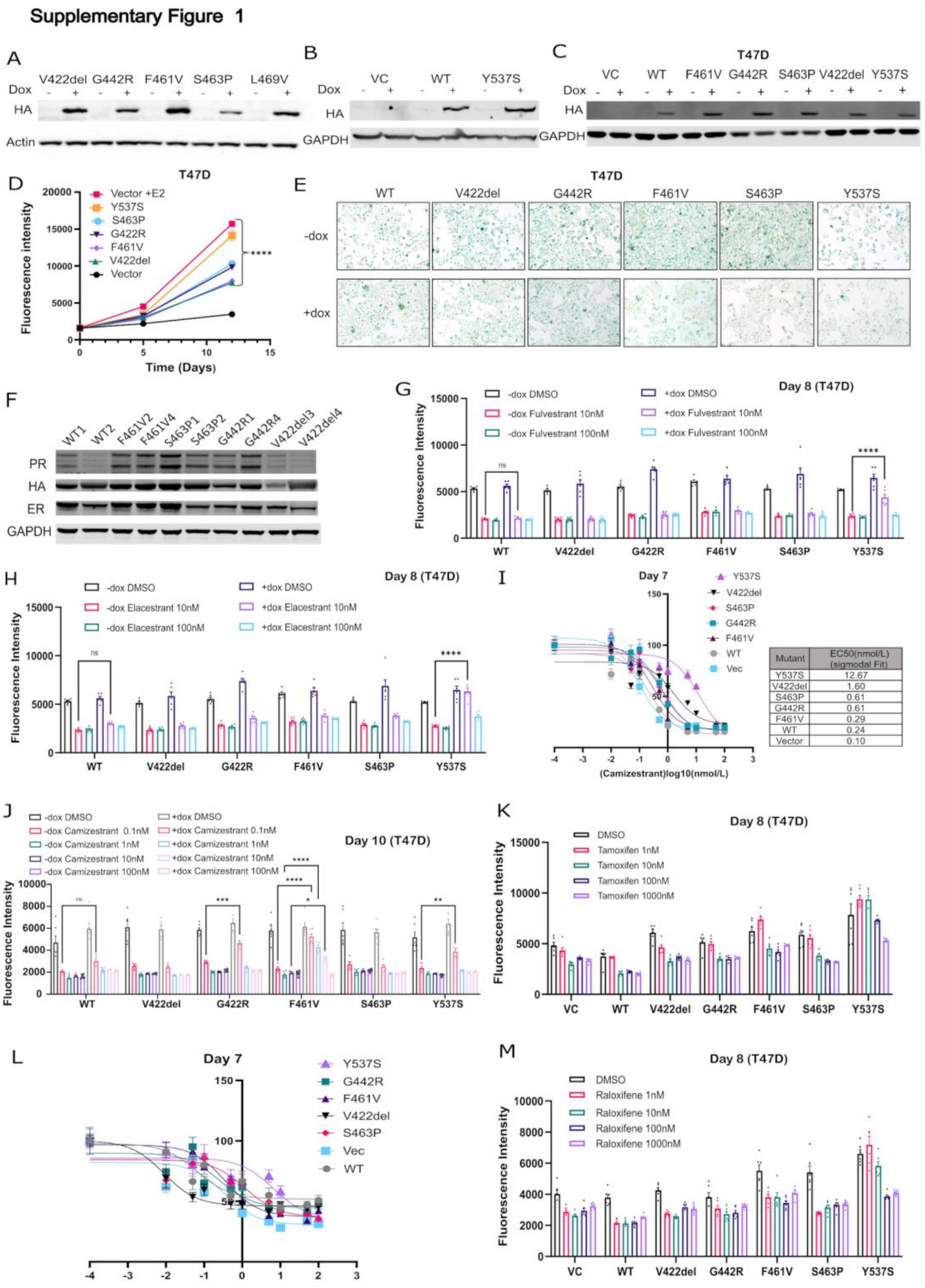

Supplementary Figure 1: Western blot showing the expression levels of HA-tagged ERα and Vector Control in MCF7 Tet-On **(A,B)** and T47D **(C)** dox-inducible cell lines grown in hormone depleted media

for 48hrs, in the presence and absence of 0.5ug/ml dox **(D)** Growth curves in T47D dox inducible cell lines grown in hormone depleted media in the presence of dox, points represent mean ± SEM (n=6), results from a 2-way ANOVA (Dunett's multiple comparison test) between each mutant and vector control for the last time point (day 12) has been shown **(E)** β-Galactosidase staining of T47D cell lines showing levels of senescence pre and post dox induction **(F)** Expression of HA-Tagged variants in tumors from Fig 1F **(G-M)** Growth inhibition of MCF7 or T47D cell lines expressing various HA-Tagged variants and vector control in varying concentrations of SERDS Fulvestrant **(G)**, Elacestrant **(H)**, Camizestrant **(I, J)**, p-value wherever stated was calculated using 2-way ANOVA with Tukey's multiple comparison test. Growth inhibition of MCF7 or T47D cell lines in varying concentrations of Tamoxifen **(K)**, Raloxifene **(L,M)**, dox concentration 0.5ug/ml, data is represented as mean ± SEM (n=6)

Supplementary Figure 2

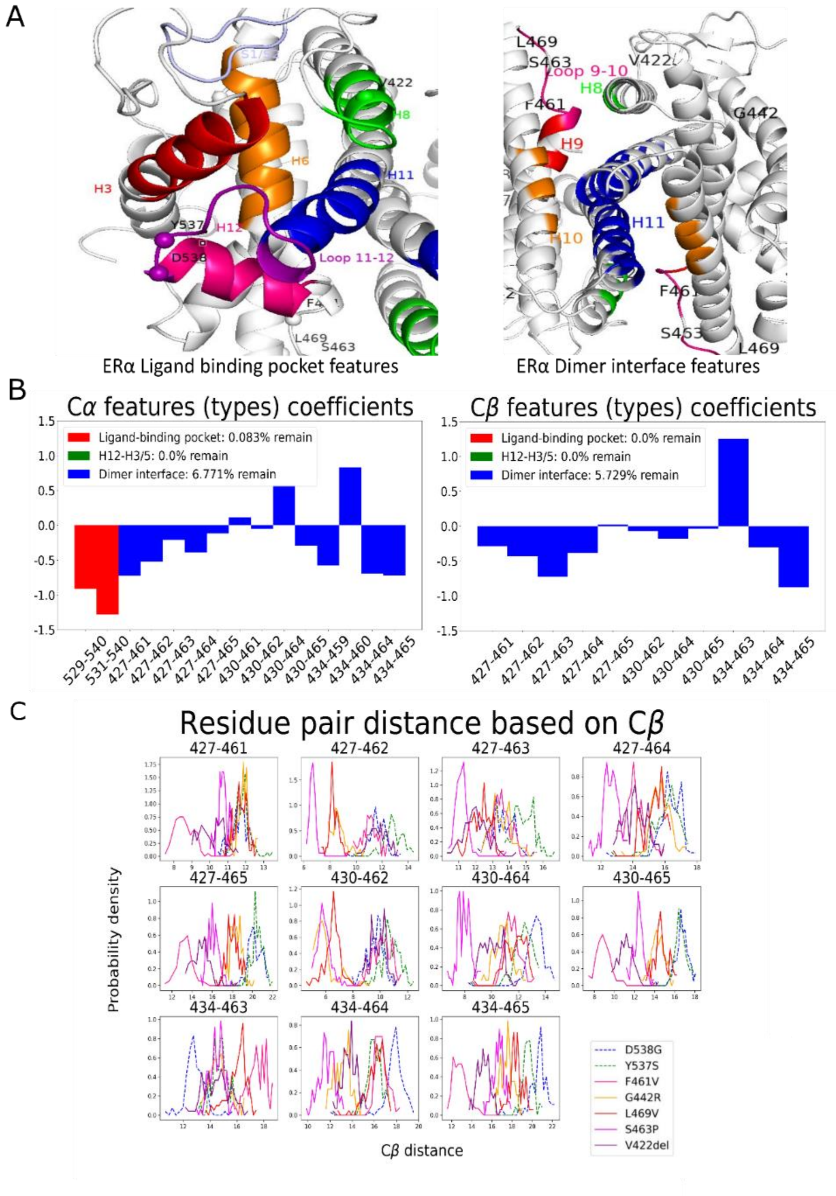

Supplementary Fig 2: (A) The residue pairs from different secondary structures in the ligand binding pocket and dimer interface are considered as a candidate feature in the machine learning model for feature selection. Left : Ligand binding pocket; residues 342 to 354 from helix 3 (red), 383 to 394 from helix 6 (orange), 402 to 410 from S1/S2 hairpin (lightblue), 418-428 from helix 8 (green), 517 to 528 from helix H11 (blue), 529 to 538 from loop between helix 11 and 12 (purple), 539 to 547 from helix 12 (hotpink). Right : Dimer interface; residues 427, 430, 434 from helix 8 (green), 455, 456, 458, 459 from helix 9 (red), 459, 460, 461, 462, 464, 465 from loop between helix 9 and 10 (hotpink) 479, 480, 483, 484, 487 from helix 10 (orange), 497, 498, 501, 502, 504, 505, 506, 508, 509, 510, 511, 512, 513, 515, 516, 519, 520, 523 from helix 11 (blue). **(B)** Machine learning (ML)-selected features, as shown in the x-labels with residue index pairs, and their coefficients. Negative coefficients suggest that two residues tend to get closer in the class II mutants and further in class I mutants. **(C)** Distributions of ML-selected pairwise Cβ distances for 7 variants in Class I (cold dashed) and Class II (warm dashed).

Supplementary Figure 3

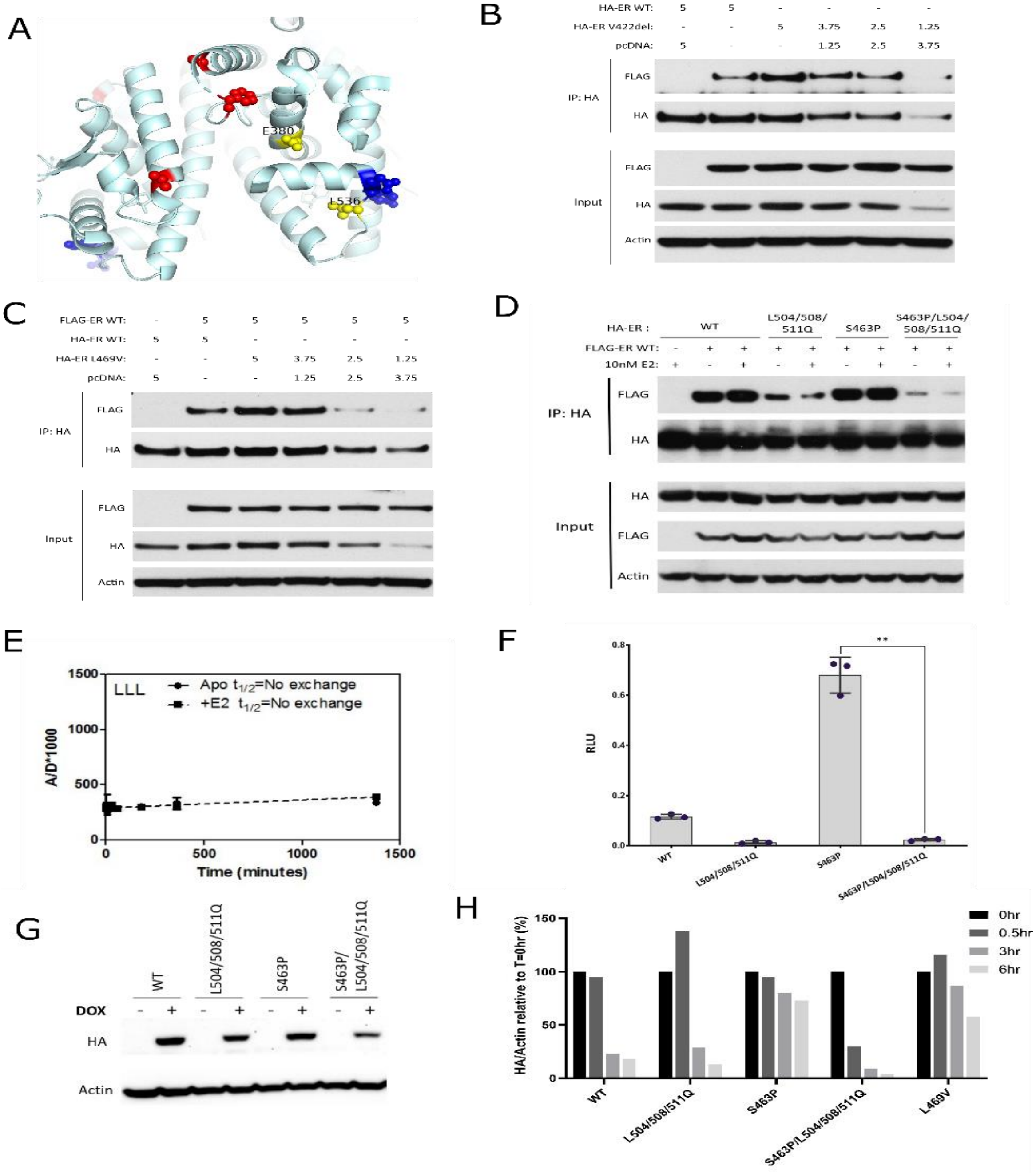

Supplementary Figure 3: (A) Structure of the ERαLBD (PDB ID: 1GWR) with the Class I residues shown in blue, class II residues in red and E380 and L536 shown in yellow. **(B)** Immunoblot of FLAG-tagged-ERα (FLAG-ERWT) that co-immunoprecipitated with HA-ER WT/V422del from MCF7 Tet-On cell lysates, prepared after co-transfecting plasmids containing a HA-ESR1 WT or the V422del variant along with FLAG-ESR1 WT. **(C)** Immunoblots from a co-immunoprecipitation experiment like (B) but transfecting HA-ESR1 L469V instead of HA-ESR1 V422del. **(D)** Immunoprecipitation of HA-tagged ERα WT/mutant from SKBR3 cell lysate transiently transfected with Flag-tagged ESR1 WT and HA-tagged ESR1 WT/mutant, grown in hormone depleted media for 48hrs post-transfection; wherever indicated 10nM E2 was added for 24hrs before harvesting. **(E)** Tr-FRET experiment as described in Fig 3B, used for ERα LBD having L504/508/511Q mutation. **(F)** Luciferase reporter assay for SKBR3 cells

co-transfected with plasmids containing the HA- ESR1 WT/mutant, ERE luciferase reporter and *Renilla* luciferase (control) reporter grown in hormone depleted media, bar graphs represent mean and error bars standard deviation from three replicates, results from a Welch's t-test have been shown. **(G)** Expression levels HA-tagged ERα of the stable cell lines from Fig 3D, after growth in hormone depleted media for 48hrs, doxycycline added to a final concentration of 0.5ug/ml where indicated. **(H)** Bar graphs represent the ratio of HA to Actin signal seen for all time points in Fig 3E, signal ratio at T=0hrs for each variant has been scaled to 100%. (Analysis performed using Image Studio Lite software)

Supplementary Figure 4

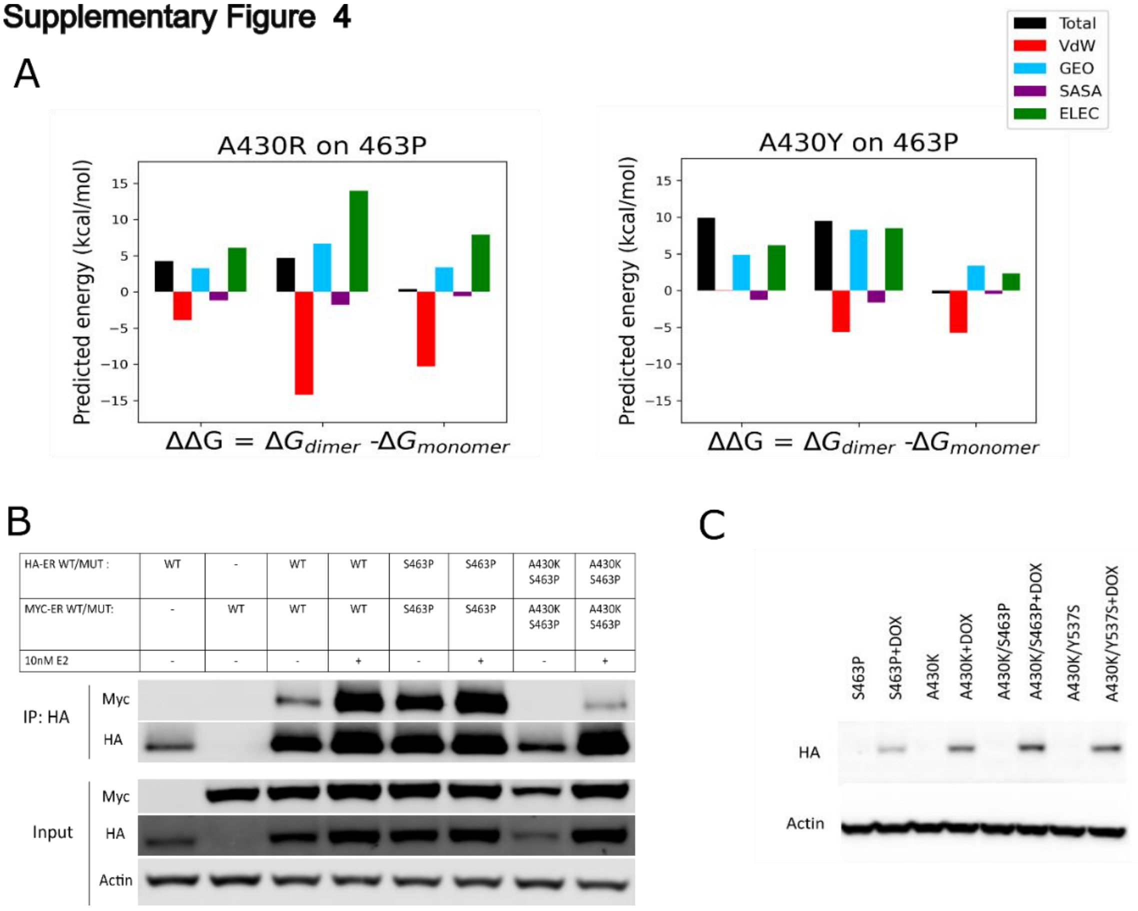

Supplementary Figure 4: (A) Two secondary mutations of A430Y and A430R to S463P was suggested by multi-state protein design method iCFN, to electrostatically weaken dimerization compared to S463P. **(B)** Immunoprecipitation of HA-ER WT/mutant from MCF7 Tet-On cell lysate after co-transfecting cells with HA-Tagged and MYC-tagged ESR1 WT or Mutant (as indicated). Growth was in hormone depleted media for 24hrs before 10nM E2 / DMSO was added following which another 24hr of growth was allowed. **(C)** Expression levels HA-tagged ERα of the stable cell lines from Fig 4D, grown in hormone depleted media for 48hrs, doxycycline is added to a final concentration of 0.5ug/ml wherever indicated.

Supplementary Figure 5

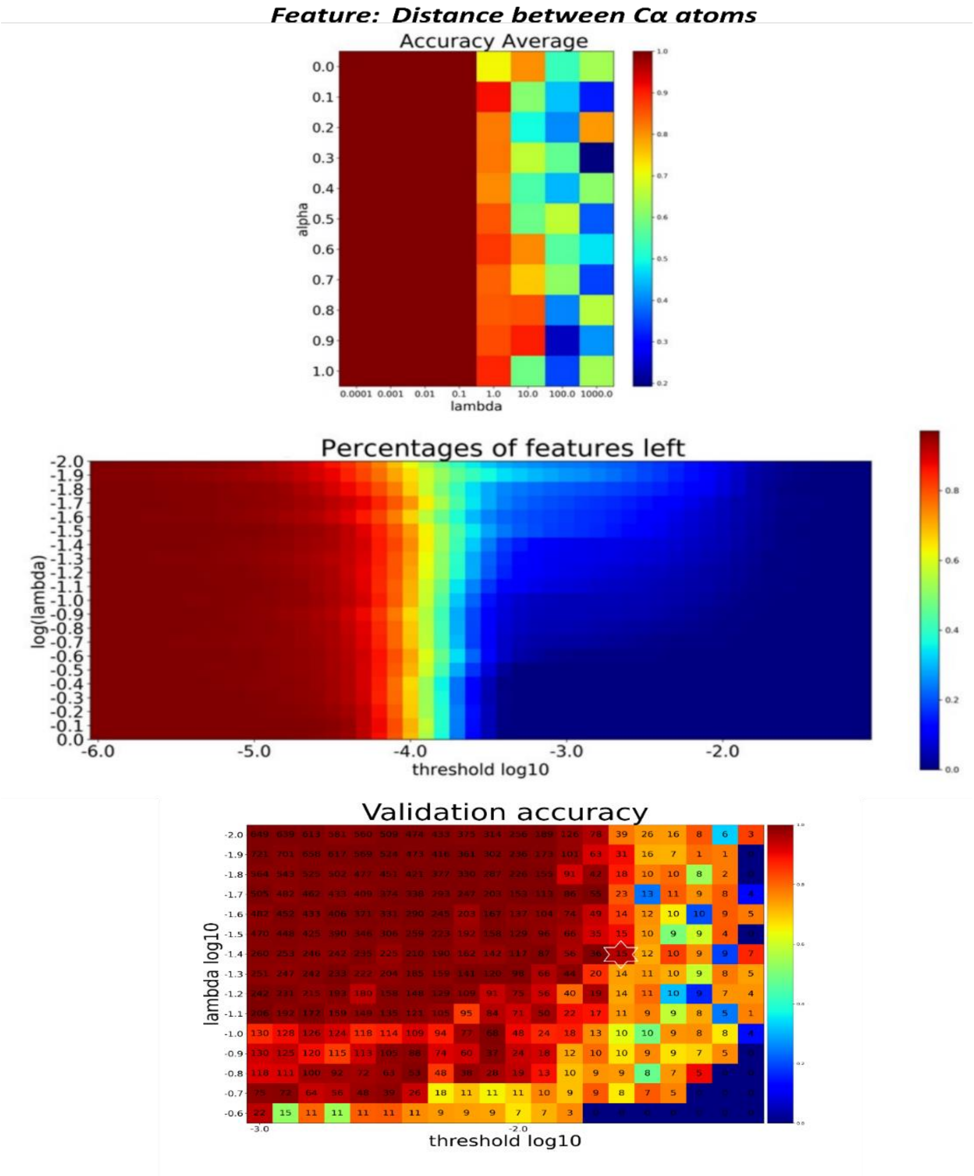

Supplementary Figure 5: Hyperparameter tuning of sparse group LASSO for the machine learning model based on Cα distances. λ controls the strength of regularization relative to classification error, whereas α balances between LASSO (for feature sparsity) and group LASSO (for feature group sparsity), the two regularization terms. (Topmost) Color-coded validation accuracy for combinations λ and α. As the validation accuracy was not sensitive to α, we fixed α at 0.5. (middle) With various λ, we remove features whose coefficient absolute values are below various thresholds, and report the percentage of remaining features as color-coded. We decide to focus on the thresholds above 1E-4 to maintain a small number of features to be selected. (Bottommost) Over combinations of λ (fine-grids between 1E-2 and 1) and the feature-selection threshold (fine-grids between 1E-4 and 1E-2), color-coded mean validation accuracy over 7 variants directed us to choose λ and the threshold at 1E-1.4 and 1E-1.6 (white star over block), respectively, by balancing a small amount of features and a high level of accuracy.

Table S1: Clinical characteristics of *ESR1* mutations

Variable	Overall N = 649	ESR1 H11/12 N = 471	ESR1 E380X N = 60	ESR1 Other N = 118	p-value[1]
**Sample Type**					<0.001
Primary	74 (12%)	37 (8.3%)	9 (15%)	28 (25%)	
Metastasis	550 (88%)	409 (92%)	51 (85%)	89 (75%)	
(Missing)	25	25	0	0	
**Sample Type Detailed**					<0.001
Treatment Naïve Primary	32 (5.1%)	7 (1.6%)	4 (6.7%)	21 (18%)	
Post-Treatment Primary/ Metastasis	592 (94.9%)	439 (98.4%)	56 (93.3%)	97 (82%)	
(Missing)	25	25	0	0	
**Receptor Status Sample**					<0.001
HR+/HER2-	540 (91%)	400 (94%)	54 (96%)	86 (75%)	
HR+/HER2+	39 (6.6%)	23 (5.4%)	2 (3.6%)	14 (12%)	
HR-/HER2+	4 (0.7%)	1 (0.2%)	0 (0%)	3 (2.7%)	
TNBC	13 (2.2%)	2 (0.5%)	0 (0%)	11 (9.7%)	
(Missing)	53	45	4	4	
**TMB (mut/Mb)**	4.9 (3.5, 7)	4.4 (3.1, 6.1)	9.7 (5.9, 15.1)	5.3 (3.5, 8.8)	<0.001

[1]Pearson's Chi-squared test; Fisher's exact test; Kruskal-Wallis rank sum test

Table S2: Inhibition of mutants by Fulvestrant (Data points and fit plotted in Fig 1G)

Cell Line	EC50 (nmol/L)(sigmodal Fit)
Y537S	8.45
V422del	0.30
G442R	0.17
F461V	0.45
S463P	0.52
Vector	0.12
WT	0.43

Table S3: Inhibition of mutants by Elacestrant (Data points and fit plotted in Fig 1H)

Cell Line	EC50 (nmol/L)(sigmodal Fit)
Y537S	1235
V422del	1.78
G442R	1.21
F461V	1.97
S463P	1.48
Vector	1.83

Table S4: Classification accuracy for each variant (test set: 100 snapshots at 90-100ns of MD) with hyperparameters chosen and parameters trained for either model.

**Mutant**	Cα model	Cβ model
Y537S	100%	100%
D538G	100%	100%
V422del	96%	99%
G442R	93%	83%
F461V	100%	100%
S463P	100%	100%
L469V	95%	94%
